# A Cell-free Expression Pipeline for the Generation and Functional Characterization of Nanobodies

**DOI:** 10.3389/fbioe.2022.896763

**Published:** 2022-04-28

**Authors:** Lisa Haueis, Marlitt Stech, Stefan Kubick

**Affiliations:** ^1^ Fraunhofer Institute for Cell Therapy and Immunology (IZI), Branch Bioanalytics and Bioprocesses (IZI-BB), Potsdam, Germany; ^2^ Institute of Biochemistry and Biology, University of Potsdam, Potsdam, Germany; ^3^ Institute of Chemistry and Biochemistry, Freie Universität Berlin, Berlin, Germany; ^4^ Faculty of Health Sciences, Joint Faculty of the Brandenburg University of Technology Cottbus—Senftenberg, The Brandenburg Medical School Theodor Fontane and the University of Potsdam, Potsdam, Germany

**Keywords:** cell-free protein synthesis, *In vitro* transcription/translation, nanobody, VHH, camelid, CHO cell lysate

## Abstract

Cell-free systems are well-established platforms for the rapid synthesis, screening, engineering and modification of all kinds of recombinant proteins ranging from membrane proteins to soluble proteins, enzymes and even toxins. Also within the antibody field the cell-free technology has gained considerable attention with respect to the clinical research pipeline including antibody discovery and production. Besides the classical full-length monoclonal antibodies (mAbs), so-called “nanobodies” (Nbs) have come into focus. A Nb is the smallest naturally-derived functional antibody fragment known and represents the variable domain (V_H_H, ∼15 kDa) of a camelid heavy-chain-only antibody (HCAb). Based on their nanoscale and their special structure, Nbs display striking advantages concerning their production, but also their characteristics as binders, such as high stability, diversity, improved tissue penetration and reaching of cavity-like epitopes. The classical way to produce Nbs depends on the use of living cells as production host. Though cell-based production is well-established, it is still time-consuming, laborious and hardly amenable for high-throughput applications. Here, we present for the first time to our knowledge the synthesis of functional Nbs in a standardized mammalian cell-free system based on Chinese hamster ovary (CHO) cell lysates. Cell-free reactions were shown to be time-efficient and easy-to-handle allowing for the “on demand” synthesis of Nbs. Taken together, we complement available methods and demonstrate a promising new system for Nb selection and validation.

## Introduction

**FIGURE 1:**
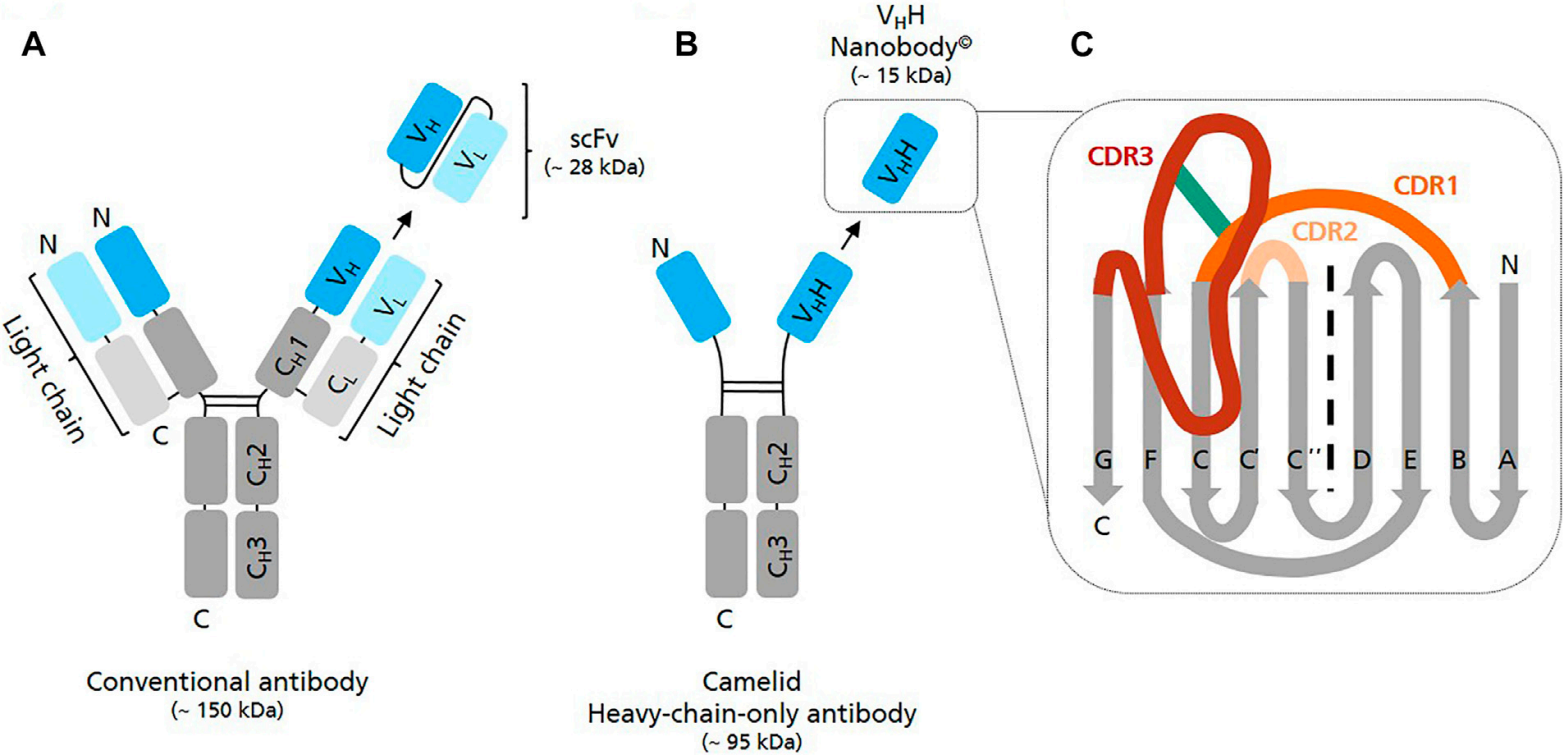
Graphical representation of different antibody structures and recombinantly expressible formats. **(A)** A conventional full length IgG antibody consists of two identical light (variable—V_L_ and constant—C_L_ domain) and two identical heavy polypeptide chains (variable V_H_ and constant—C_H_1/2/3 domains) which assemble into a heterotetramer. Single chain variable fragments (scFv) are recombinant binders consisting of the variable domain of antibody heavy chain (V_H_) and antibody light chain (V_L_) fused by a flexible peptide linker. **(B)** A camelid heavy-chain-only antibody (HCAb) consists of two identical heavy polypeptide chains (variable—V_H_H and constant C_H_2/3 domains) forming a homodimer. The antigen binding region of HCAbs is called V_H_H domain or Nanobody. **(C)** Schematic view of the V_H_H domain consisting of 2 ß-sheets, one with 4 and one with 5 ß-strands. Scaffold ß strands are depicted in grey (A, B, E, D, C″, C′, C, F, G), antigen-binding loops in dark orange (CDR1), light orange (CDR2) and in red (CDR3). The frequently observed interloop disulfide bond betwenn CDR1 and CDR3 is depicted in green.

Nanobodies (Nbs) have a molecular mass of approximately 12–15 kDa and are the smallest natural antigen-binding entities known so far (Genst et al., 2006). They originate from unique functional heavy-chain-only antibodies (HCAbs) circulating in the blood of Camelidae*.* Unlike conventional antibodies which consist of two identical heavy and two identical light chains forming a heterotetramer, HCAbs are devoid of light chains and consist exclusively of two heavy chains which assemble to a homodimer (Hamers-Casterman et al., 1993) (Figure 1). The variable antigen binding domain of these HCAbs, the so-called V_H_H domain or Nb, can be recombinantly expressed as separate entity, while retaining full antigen binding capacity (van der Linden et al., 1999). Whereas the term nanobody^©^ is the registered trademark of Ablyx (Ghent, Belgium), V_H_Hs are also known as single-domain antibodies (sdAbs).

In comparison to other monomeric binders such as affibodies, monobodies or anticalins Nbs have the advantage that they originate from naturally-occurring antibodies whose genes have evolved over millions of years (Flajnik et al., 2011). Accordingly, Nbs are extremely robust, highly soluble and strictly monomeric thereby representing well optimized high-affinity binders (Muyldermans 2013). In comparison to single-chain variable fragments (scFv), which are also relatively small binders (∼25 kDa) but derived from conventional antibodies (∼160 kDa), they are less prone to denaturation/aggregation and spontaneous dimerization (Muyldermans 2021a).

Nbs have found numerous applications as research tools, in diagnostics and also therapeutics (Muyldermans 2021b). For example, Nbs contributed to the elucidation of multiple G protein coupled receptor (GPCR) structures, as they can bind conformational and non-linear epitopes better than conventional antibodies (Rasmussen et al., 2011; Groof et al., 2019). Further, Nbs have been shown to be excellent tools for the generation of intrabodies targeting intracellular structures (reviewed in (Soetens et al., 2020; Wagner and Rothbauer 2020). Nb-based intrabodies have been used as imaging probes (Rothbauer et al., 2006; Traenkle and Rothbauer 2017), biosensors (Irannejad et al., 2013; Stoeber et al., 2018; Cao et al., 2019) and even as intracellular modulators of signaling pathways and cellular targets (van Impe et al., 2013; Gulati et al., 2018; Singh et al., 2018) (reviewed in (Wagner and Rothbauer 2020). Moreover Nbs have been shown to be well-suited for *in vivo* imaging approaches as they show superior properties with regard to tissue distribution, rapid tumor accumulation, tumor penetration and fast clearance from the blood circulation (Huang et al., 2008; Chanier and Chames 2019). One example is a^68^Ga-labeled HER2-targeting Nb which is in clinical trials for imaging of HER2 in breast cancer patients (Keyaerts et al., 2016). Caplacizumab is the first EMA and FDA-approved Nb specific for von Willebrand factor for the treatment of acquired thrombotic thrombocytopenic purpura (Callewaert et al., 2012; Duggan 2018; Scully et al., 2019).

Due to their small size and the lack of posttranslational modifications, Nbs are easy to tailor and can be cost-efficiently produced in microbial systems including *Escherichia coli* (*E. coli*) and yeast with high yields (reviewed in (Marco 2020)). In the last decade, cell-free translation systems have become popular as they allow for the efficient, versatile and high-throughput-compatible synthesis of a wide variety of target proteins *in vitro* and independently of living cells (Brookwell et al., 2021). In addition, cell-free systems have demonstrated great potential for the *in vitro* evolution of proteins and in particular the selection of antibodies based on *in vitro* display technologies such as mRNA (Roberts and Szostak 1997), cDNA (Yamaguchi et al., 2009) and ribosome display (Hanes and Plückthun 1997; He and Taussig 1997). *In vitro* display based on *E. coli* (Doshi et al., 2014; Chen et al., 2021), rabbit reticulocyte (Fenderico et al., 2019) and *Leishmania torentolae* (Bencurova et al., 2015) cell-free systems has already demonstrated its feasibility for Nb enrichment and identification. Using a eukaryotic cell-free system for *in vitro* translation and evolution of proteins is tempting because these systems are known for a lower RNase activity and superior capacities to fold and functionally display eukaryotic proteins (Hanes et al., 1999). For these reasons we were highly encouraged to probe the capabilities of the previously published mammalian cell-free system based on translationally active Chinese hamster ovary (CHO) cells (Brödel et al., 2013; Brödel et al., 2014) for Nb synthesis *in vitro*. Cell-free synthesis of conventional antibodies and scFv fragments was already reported by using this system; however, functionally active binders were only obtained upon their signal-peptide induced translocation into the lumen of the CHO lysate-contained endogenous microsomes (Stech et al., 2017).

The aim of the present study was to investigate first, if functionally active Nbs could be synthesized within the CHO cell-free system at all and second, to analyze if the translocation step was required to achieve functionality. As model proteins, we chose two Nbs, one being specific for human epidermal growth factor receptor (EGFR) (Schmitz et al., 2013), while the other one was recognizing green fluorescent protein (GFP) (Kubala et al., 2010). In order to find the best conditions for cell-free Nb synthesis with regard to protein yield and functionality, we analyzed each Nb with and without melittin signal sequence and investigated the Nbs in the “cytosolic” fraction of the lysate, which comprised the non-translocated Nbs, and in the microsomal fraction, comprising co-translationally translocated Nbs. Importantly, our results show that the Nbs we analyzed did not require a translocation step to achieve binding activity. Thus, we present the CHO cell-free system as novel platform for the “on demand” synthesis and analysis of soluble and functionally active Nbs, thereby providing a time-saving and convenient alternative to classical cell-based production.

## Materials and Methods

### Template Generation

In this study two different Nbs known from the literature were used as models to analyze the feasibility of the CHO cell-free system for Nb synthesis and functional analysis. First, a Nb specific for GFP was chosen as model. This Nb is known to bind specifically to GFP and YFP, but without affecting the fluorescence of these reporter proteins (Kubala et al., 2010). Secondly, an EGFR-specific Nb (7D12) was chosen according to Schmitz et al. (2013). V_H_H domain 7D12 is reported to bind to EGFR domain III and to block ligand binding to EGFR similar to the monoclonal antibody cetuximab (Roovers et al., 2011; Schmitz et al., 2013). Coding sequences of the anti-GFP-Nb and the anti-EGFR-Nb were codon-optimized for *Cricetulus griseus* and *homo sapiens*, respectively, and equipped with the regulatory sequences necessary to enable *in vitro* transcription and translation according to Stech et al., 2017 (5′ untranslated region (UTR): T7 promotor sequence, multiple cloning site (MCS), internal ribosomal entry site (IRES) from the intergenic region (IGR) of the Cricket paralysis virus (CrPV), GCT as start codon; 3′ UTR: T7 terminator sequence, MCS) (Stech et al., 2017). Different templates were generated per Nb candidate: One with melittin signal sequence (MKFLVNVALVFMVVYISYIYAD (Tessier et al., 1991)), designated NCM-anti-GFP-Nb and NCM-anti-EGFR-Nb, and the other without melittin signal sequence, designated NC-anti-GFP-Nb and NC-anti-EGFR-Nb. The signal sequence of honey bee mellittin was chosen, as it has been shown before to allow for efficient translocation into microsomal vesicles in various cell-free systems (Brödel et al., 2013). Further, Nb sequences were C-terminally fused to a c-Myc and 6x-His-Tag sequence to enable detection by anti-c-Myc monoclonal antibodies and for purification purposes, respectively. DNA templates were synthesized *de novo* and cloned into appropriate vectors (pUC57-1.8k) by Biocat GmbH (Biocat GmbH, Heidelberg). Plasmid preparations for cell-free protein synthesis were prepared using the PureLink®HiPure Plasmid Midiprep Kit (Thermo Fisher Scientific, Waltham, United States) according to the manufacturer’s instructions and subsequently control digested as well as sequenced to verify the correct DNA sequence (LGC Genomics GmbH, Berlin, Germany).

### Cell-free Protein Synthesis

Coupled transcription-translation reactions were performed according to (Brödel et al., 2013; Thoring and Kubick 2018). In brief, translation reactions were composed of 40% (v/v) S7 nuclease-treated CHO lysate containing endogenous microsomal vesicles originating from the ER, HEPES-KOH (pH 7.6, f.c. 30 mM, BioMol GmbH, Hamburg, Germany), complete amino acids (f.c. 100 µM), Mg(OAc)_2_ (f.c. 3.9 mM), KOAc (f.c. 135 mM, Merck, Darmstadt, Germany), spermidine (f.c. 0.25 mM, Sigma-Aldrich, St. Louis, United States), energy components (f.c. 1.75 mM ATP, f.c. 0.3 mM GTP, f.c. 0.3 mM CTP, f.c. 0.3 mM UTP), 0.1 mM (f.c.) m7G(ppp)G (Prof. Edward Darzynkiewicz, Warsaw University, Poland), creatine phospate (f.c. 20 mM), T7 polymerase (f.c. 1 U/µl) (Agilent Technologies, Santa Clara, United States) and ^14^C-leucine (f.c. of 30 μM; specific radioactivity 46.15 dpm/pmol (PerkinElmer LAS (Germany) GmbH, Rodgau, Germany) in order to allow for the subsequent quantitative and qualitative analysis of cell-free synthesized proteins. Protein synthesis was initiated by addition of DNA template (f.c. 60 ng/μL). Reactions were incubated for 3 h at 30°C at 600 rpm in a standard thermomixer (Eppendorf Thermomixer Comfort). Background translational activity was monitored by performing a translation reaction without supplementation of plasmid, called “no template control” (NTC).

CHO lysates containing endogenous microsomal vesicles derived from the ER were prepared as described previously (Brödel et al., 2013; Thoring and Kubick 2018). In order to promote disulfide bond formation within the cell-free system Dithiothreitol (DTT) which is usually added at 4 mM f.c. was omitted from the lysate preparation procedure. In brief, CHO cells were grown exponentially in well-controlled fermenters at 37°C up to 18 × 10^6^ cells/ml using chemically defined, serum-free media (PowerCHO^TM^ 2 CD Medium, Lonza, Basel, Switzerland). After harvesting the cells by centrifugation at 200 × g for 15 min, pellets were washed twice and resuspended in buffer containing 30 mM HEPES-KOH (pH 7.5) and 100 mM NaOAc. Subsequently, the cell suspension was passed through a 20-gauge needle using a syringe which resulted in the mechanical disruption of the cells. Nuclei and cell debris were removed by a centrifugation step at 6,500 × g for 10 min. The obtained supernatant was subjected to a gel filtration step using Sephadex G-25 column (GE Healthcare, Freiburg, Germany) equilibrated in a buffer containing 30 mM HEPES-KOH (pH 7.5) and 100 mM. Elution fractions (1 ml each) with an RNA content above an absorbance of 100 at 260 nm were pooled. In order to remove endogenous mRNA, cell lysates were treated with S7 micrococcal nuclease (Roche, Mannheim, Germany) (f.c. 10 U/ml) and CaCl_2_ (f.c. 1 mM) and incubated for 20 min at room temperature (RT). Micrococcal S7 nuclease was inactivated by adding EGTA (f.c. 6.7 mM) and CHO lysates were further supplemented with creatine kinase (f.c. 100 μg/ml). Lysates were shock frozen in liquid nitrogen and subsequently stored at −80°C until further usage.

After completing the translation reaction, samples were centrifuged at 16,000 × g for 10 min at 4°C in order to separate the microsomes from the soluble fraction of the translation mixture. The resulting supernatant (designated as SN1) was transferred to a fresh reaction tube and stored on ice until further analysis, while the microsomal pellet was resuspended in 1x PBS containing 0.2% n-Dodecyl-β-D-Maltoside (DDM) in order to enable release of translocated and microsome-contained Nbs. Microsomes were resuspended manually by repeated up and down pipetting followed by vortexing and shaking on a vibrax for approximately 45 min. To separate the released proteins from microsomal membrane remnants, a second centrifugation step was performed. The resulting supernatant (designated as SN2) was transferred to a fresh reaction tube and stored on ice until further analysis. Reactions supplemented with ^14^C-leucine were analyzed by SDS-PAGE followed by autoradiography and liquid scintillation counting, while non-radioactive samples were subjected to functional analysis by enzyme-linked immunosorbent assay (ELISA).

### SDS-PAGE and Autoradiography

5 µL aliquots of SN1 or SN2 were mixed with 45 µL ddH_2_O and 150 µL pre-cooled acetone. Samples were incubated for a minimum of 15 min on ice and subsequently centrifuged at 16,000 × g and 4°C for 10 min. The resulting supernatant was discarded and the protein pellet was dried for at least 30 min at 45°C and resuspended in 20 µL 1x LDS sample buffer (NuPAGE™ LDS sample buffer (f.c. 1x), Thermo Fisher Scientific, Waltham, United States) supplemented with 50 mM (f.c.) DTT (Roche). Samples were heated for 10 min at 70°C and analyzed by sodium dodecylsulfate (SDS) polyacrylamide gel electrophoresis (PAGE) using self-made 14% Tris-Glycin gels (SureCast Gel Handcast System, Thermo Fisher Scientific, Waltham, United States) and Tris-Glycin running buffer. The electrophoretic separation was conducted at 155 V for 65 min. SDS-PAGE gels were subsequently stained using Coomassie blue solution (SimplyBlue SafeStain, Thermo Fisher Scientific, Waltham, United States) and dried on Whatman paper for 70 min at 70 °C (Unigeldryer, 3545D, Uniequip Laborgerätebau-und Vertriebs GmbH, Planegg, Germany). ^14^C-leucine-labeled protein bands were visualized using the Amersham RGB Imager (GE healthcare) after incubation of dried gels for at least three days on a phosphor screen (GE healthcare).

### Quantitative Protein Analysis

Protein yields of *de novo* synthesized ^14^C-leucine labeled Nbs were determined by liquid scintillation counting as described previously (Stech et al., 2012). In brief, 5 µL aliquots of SN1 or SN2 were precipitated in 3 ml of 10% (v/v) TCA—2% (v/v) casein hydrolysate (Carl Roth GmbH, Karlsruhe, Germany), boiled for 15 min at 80°C and subsequently cooled on ice for at least 30 min. Protein solutions were filtered using a vacuum filtration system (Hoefer, Holliston, United States) and concentrations of ^14^C-leucine labeled proteins which were retained on the membrane filters (VWR, Darmstadt, Germany) were calculated based on liquid scintillation counting using the Hidex SL600 scintillation counter.

### ELISA

Binding activities of cell-free synthesized Nbs in translation mixture fraction SN1 and SN2 were analyzed by ELISA. For the analysis of the anti-EGFR-Nb 96-well microtitre plates (Greiner Bio-one, Kremsmünster, Austria) were coated over night at 4°C with 50 µL/well recombinant EGFR extracellular domain (ECD) (f.c. 0.7 μg/ml in 1x PBS, pH 6). Recombinantly expressed EGFR ECD was purchased from Acro biosystems (Newark, United States) (EGFR, ECD L25-S645, His-Tag, 70.5 kDa, ABIN6253725). Accordingly, 96-well microtitre plates were coated with 50 µL/well of recombinant GFP (f.c. 1.3 μg/ml in sodium carbonate buffer, pH 9.6) for the analysis of the anti-GFP-Nb. Recombinant GFP-GST was kindly provided by the preclinics Gesellschaft für präklinische Forschung mbH (Potsdam, Germany). Subsequently, ELISA plates were washed 3x using 1x PBS-T containing 0.05% Tween-20 (200 µL/well). For the anti-EGFR-Nb all of the applied buffers were adjusted to pH 6, while for the anti-GFP-Nb the pH was adjusted to 7.4. Plates were blocked with 1xPBS containing 2% BSA (200 µL/well) for 2 h at RT and 250 rpm. After washing the plates 3x with washing buffer, wells were incubated for 2 h at RT and 250 rpm with translation mixture fractions SN1 and SN2 containing cell-free synthesized Nbs in serial dilutions (50 µL/well) using 1x PBS containing 1% BSA for SN1 and 1x PBS containing 1% BSA and 0.2% DDM for SN2 as dilution buffer. Plates were washed again 3x and incubated with mouse anti-c-Myc-tag antibody (Life technologies, Carlsbad, United States) diluted 1:1000 in PBS containing 1% BSA in (50 μL/well) for 2.5 h at RT and 250 rpm. Upon washing the plates for 3x, plates were incubated with anti-mouse-IgG, HRP-linked antibody (Cell signaling, Danvers, United States and advansta, San Jose, United States) diluted 1:3000 in PBS containing 1% BSA (50 μL/well) for 1 h at RT and 250 rpm. After washing 3x, bound antibodies were detected by adding TMB substrate solution (Life technologies, Carlsbad, United States) (50 µL/well). Color development was stopped by addition of 0.5 M H_2_SO_4_ (50 μL/well) after incubating for 10–15 min Absorbance was measured at 450 and 620 nm (as reference) using the FLUOstar Omega (BMG Labtech, Ortenberg, Germany).

## Results

In order to analyze the influence of a signal peptide induced translocation of *de novo* synthesized Nbs to the lumen of the endogenous microsomes contained in the CHO lysate, coding sequences of anti-GFP-Nb and anti-EGFR-Nb with melittin signal sequence (NCM-anti-GFP-Nb/NCM-anti-EGFR-Nb) and without signal sequence (NC-anti-GFP-Nb/NC-anti-EGFR-Nb) were compared regarding Nb yield and functionality. Nb yield and binding activity were analyzed in two different preparations of the completed cell-free reaction, designated as SN1 and SN2. Nbs which have not been translocated to the microsomes, either because of the lack of a signal sequence (NC constructs) or because of insufficient translocation, were expected in translation mixture fraction SN1, while Nbs which have been translocated to the lumen of the microsomes were expected in fraction SN2 upon detergent-mediated microsome disintegration.

**FIGURE 2:**
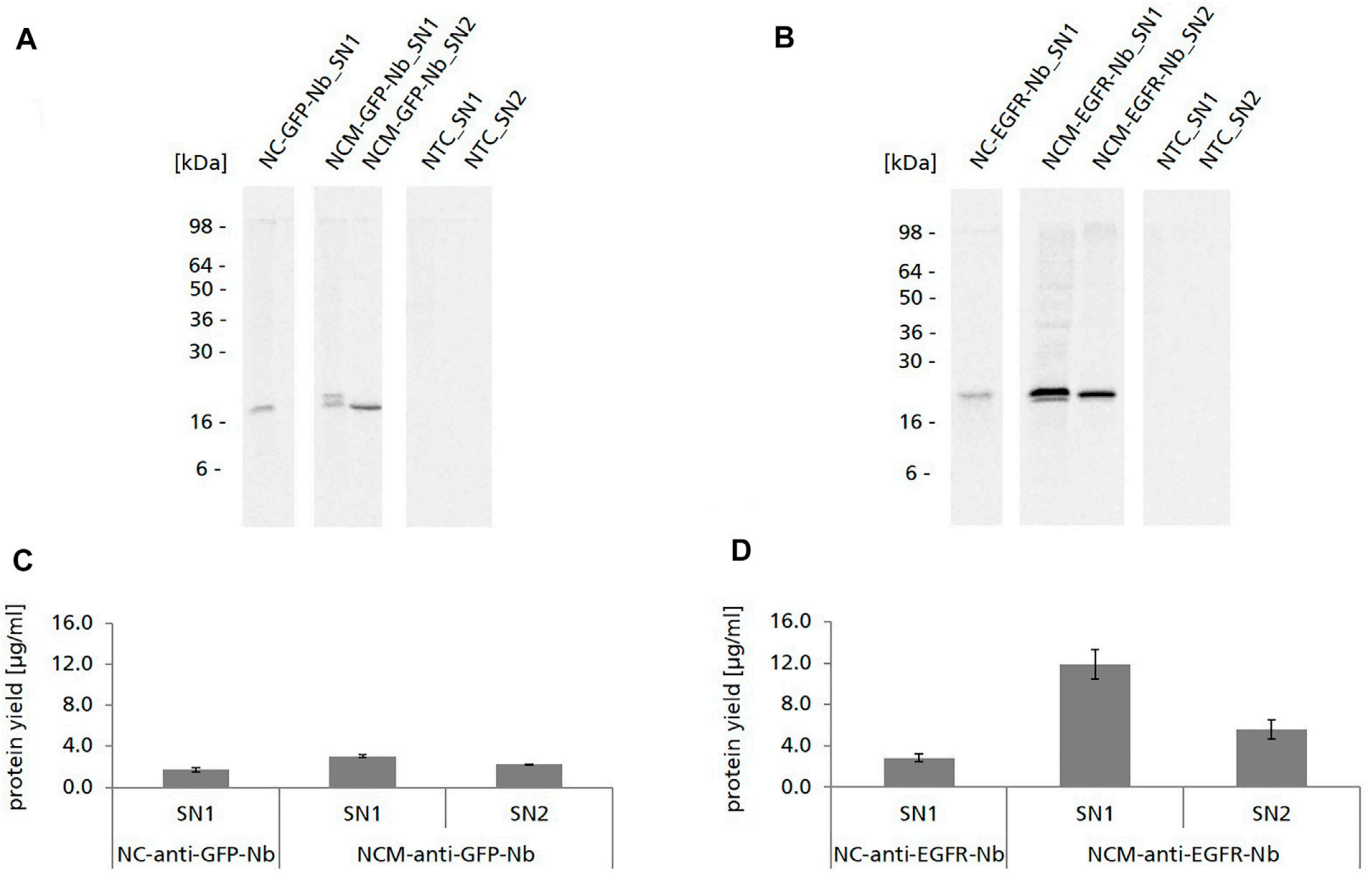
Cell-free synthesis of anti-GFP-Nb and anti-EGFR-Nb based on CHO lysate. **(A,B)** Autoradiographs showing ^14^C-leucine labeled Nanobodies in both soluble fractions of the translation mixture (SN1, SN2). **(C,D)**. Quantitative analysis of cell-free synthesized Nbs was performed by liquid scintillation counting. Standard deviations were calculated from quadruplicate analysis.

Nb constructs without signal sequence were detected as one distinct protein band in the autoradiograph showing the expected molecular mass (∼15 kDa for NC-anti-GFP-Nb, ∼18 kDa for NC-anti-EGFR-Nb) (Figures 2A,B). Nb constructs with signal sequence (NCM-anti-GFP-Nb/NCM-anti-EGFR-Nb) were detected as double band in SN1, while in SN2 only one band appeared which was on the level of the lower band of SN1. To exclude glycosylation as a reason for the observed double band, samples were digested with endoglycosidase H (Endo H) and Peptide N-glycosidase F (PNGase F) and analyzed by autoradiography (data not shown). As expected, the double band did not disappear by the digestion suggesting that it represented a mixture of Nb species with signal sequence and without, probably as a consequence of incomplete signal peptide cleavage within the cell-free system. The melittin signal peptide has a molecular mass of 2.5 kDa which corresponded to the band shift detected in the autoradiograph (Figures 2A,B).

For both Nbs we observed significantly higher protein yields when using the constructs with melittin signal sequence in comparison to the constructs without signal sequence (∼3 μg/ml NCM-anti-GFP-Nb and ∼12 μg/ml NCM-anti-EGFR-Nb *vs*. ∼1.7 μg/ml NC-anti-GFP-Nb and ∼2.9 μg/ml for NC-anti-EGFR-Nb) (Figures 2C,D). Strikingly, protein yields of the anti-GFP-Nb were considerably lower compared to the yields of the anti-EGFR-Nb. In general, protein yields of the anti-GFP-Nb were lower than expected for the CHO cell-free system which is reputed for its high productivity (Thoring et al., 2017). Interestingly, also different optimization strategies such as variation of the template concentration, incubation temperature and time as well as the addition of supplements (PolyG) did not result in considerably higher protein yields for this particular Nb (data not shown).

**FIGURE 3:**
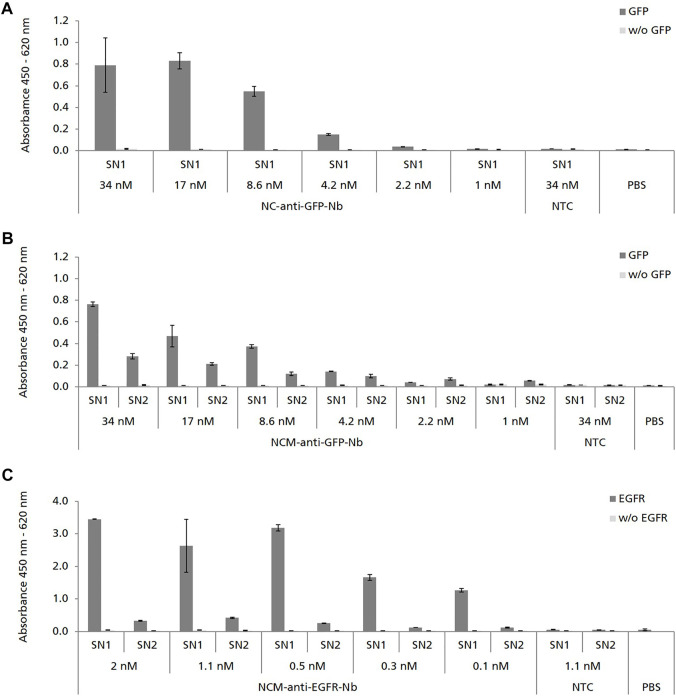
ELISA analysis showing the specific binding of cell-free synthesized Nbs to their corresponding antigens. Nbs without signal sequence (NC) were analyzed in SN1 (comprising non-translocated Nbs). Nbs with signal sequence (NCM) were analyzed in SN1 and additionally in SN2 (comprising Nbs which were solubilized from the lumen of the endogenous CHO microsomes). No template control (NTC) and PBS were analyzed in parallel as negative controls. To monitor background binding Nb samples were also analyzed on wells coated without (w/o) antigen. **(A,B)** Binding of anti-GFP-Nb to its antigen green fluorescent protein (GFP) was analyzed in serial dilutions in the range of 34–1 nM. **(C)** Binding of anti-EGFR-Nb to epidermal growth factor receptor (EGFR) was analyzed in serial dilutions in the range of 2–0.1 nM. Standard deviations were calculated from quadruplicate analysis **(A,B)** and duplicate analysis **(C)**.

We next analyzed the binding capabilities of cell-free synthesized Nbs by using ELISA. NC-anti-GFP-Nb was analyzed in SN1, while NCM-anti-GFP-Nb was analyzed in both, SN1 and SN2 in serial dilutions starting at 34 nM cell-free synthesized Nb. ELISA analysis revealed specific binding of NC-anti-GFP-Nb and NCM-anti-GFP-Nb in SN1 to its antigen GFP (Figures 3A,B). The binding signal was shown to be dependent on the applied Nb concentration as the OD values decreased with increasing dilution of the Nb sample. Although applied in the same molar concentration NCM-anti-GFP-Nb in SN2 showed a lower binding signal compared to NCM-anti-GFP-Nb in SN1 (Figure 3B).

Due to the observation that higher protein yields were observed for the constructs with melittin signal sequence, NCM-anti-EGFR-Nb was preferred over NC-anti-EGFR-Nb (Figure 2D). Consistent with the ELISA data obtained for NCM-anti-GFP-Nb, melittin signal peptide fused anti-EGFR-Nb showed a concentration-dependent specific binding signal in SN1, while a lower signal was observed for SN2 (Figure 3C).

Taken together, our results demonstrate the specific binding of cell-free synthesized, melittin signal peptide fused anti-GFP-Nb and anti-EGFR-Nb to their corresponding antigens. Although the presence of the signal sequence was not mandatory for Nb functionality, it was shown to increase the synthesis efficiency.

## Discussion

In view of the therapeutic potential of Nbs and upcoming strategies to further functionalize Nbs eukaryotic selection and production systems are of highest relevance. Mammalian systems in particular are more demanding and expensive, and therefore their application for Nb expression must be justified. This is for example the case if the Nb is fused to the Fc part of IgG antibodies to restore ADCC (antibody dependent cell-mediated cytotoxicity) and CDC (complement*-*dependent cytotoxicity) effector functions *in vivo* requiring glycosylation of N297 in the C_H_2 domain and to increase affinity by avidity (Bobkov et al., 2018; Godakova et al., 2019). In addition, the generation of Nbs fused to horse radish peroxidase (HRP) may justify the use of a mammalian expression system, since HRP is known to be difficult-to-express in bacteria (Sheng et al., 2019; Marco 2020). A further indication can be seen when Nbs are produced as fusions with proteinacous toxin moieties (Yu et al., 2017) which might demand for an eukaryotic and even mammalian expression system. Further, the development of intrabodies requires efficient expression of Nbs in mammalian systems as intrabodies must be able to maintain functionality even when they are located in the reducing environment of the cytoplasm (Wagner and Rothbauer 2020). In order to meet the demand for efficient mammalian Nb selection and production systems, we focused on the well-established CHO cell-free system (Brödel et al., 2013; Thoring et al., 2016). As previously reported, the CHO cell-free system allows for the folding and assembly of complex proteins (Thoring et al., 2017; Zemella et al., 2019) and protein multimers (Thoring et al., 2017; Ramm et al., 2020; Ramm et al., 2021) and enables several types of posttranslational modifications on target proteins, such as N-glycosylation (Thoring and Kubick 2018), lipidation (Fenz et al., 2014), phosphorylation (Thoring et al., 2016) and disulfide bridge formation (Martin et al., 2017; Stech et al., 2017). Though Nbs are not posttranslationally modified, except for their conserved intradomain disulfide bridge, the CHO cell-free system offers the advantages of cell-free systems in general: speed, open reaction design, high-throughput and on-demand synthesis (Thoring et al., 2016). At the same time, it provides a eukaryotic folding apparatus, which can be beneficial for diverse applications, such as the *in vitro* evolution of Nb fusion proteins and intrabodies as described above.

Here we show the proof-of-concept that functional Nbs can be synthesized within a 3 h incubation time by using the cell-free CHO system. As far as we know this is the first time that functional Nbs have been synthesized in a eukaryotic cell-free system apart from rabbit reticulocyte and *Leishmania* systems. The cell-free CHO system was capable of producing the selected model Nbs in yields up to ∼12 μg/ml (Figure 2) which was sufficient for a straight-forward functional analysis of Nbs by ELISA, directly following cell-free synthesis and without requiring a purification step. In comparison, scFv and Fab fragments have been produced in yields of 50–200 μg/ml in a cell-free *E. coli* system (Wu et al., 2022). Unlike *E. coli*, the cell-free reticulocyte system is related to rather low protein yields (Carlson et al., 2012; Gregorio et al., 2019). However, reticulocyte lysate systems are based on an ethically questionable preparation strategy as the lysates are prepared from rabbits which have been made anemic and bled by heart puncture (Darbnbrough et al., 1973). In contrast, the mammalian cell-free system used here is prepared from the immortalized CHO-K1 cell line which can be cultivated in bioreactors using chemically defined and serum-free media, underlying a very strict process control (Thoring and Kubick 2018; Brödel et al., 2013). Based on the results we obtained by ELISA (Figure 3) the EC_50_ value of cell-free synthesized NCM-anti-EGFR-Nb analyzed in SN1 was estimated to be in the lower nanomolar range which corresponded to the data obtained for the cell-based produced Nb (Gainkam et al., 2008; Roovers et al., 2011). However, binding activities of cell-free versus cell-based produced Nbs were not compared side-by-side, which does not allow for a valid conclusion on that point.

The CHO cell-free system used in this study contained endogenous microsomal vesicles derived from the ER of the cells used for lysate preparation. By using an appropriate signal sequence target proteins can be co-translationally translocated to the lumen of these microsomes or in the case of membrane proteins embedded into the lipid bilayer (Fenz et al., 2014). In this study we found that the fusion of the melittin signal sequence to the Nb led to translocation to the lysate-contained microsomes. However, translocation was not mandatory for Nb functionality in contrast to scFv and IgG antibody formats synthesized in a *Spodoptera frugiperda* 21 (*Sf*21) and CHO cell-free system (Stech et al., 2017; Stech et al., 2014). In fact, translocated and microsome released Nbs were found to be less active compared to non-translocated Nbs (Figure 3). On the other hand the fusion to the signal sequence exhibited a positive effect on the protein yields of cell-free synthesized Nbs (1.8-fold for anti-GFP-Nb and 4-fold for anti-EGFR-Nb in comparison to the Nb without signal sequence) (Figure 2). It is known that signal sequences can stimulate protein synthesis in *E. coli* cell-free systems (Ahn et al., 2006) and the same observation has been made for a microsome-containing *Sf*21 cell-free system (Sonnabend et al., 2015). We provide evidence that this holds true also for the CHO cell-free system. It can be speculated that the presence of the melittin signal sequence at the 5′ end of the Nb gene stabilized translation initiation and translational processivity, which resulted in increased protein yields.

In previous reports, the CHO cell-free system has been shown to enable N-glycosylation of target proteins, which frequently resulted in the detection of glycosylated and non-glycosylated protein species (Brödel et al., 2014; Thoring et al., 2016). However, Nbs in general were not expected to be glycosylated and accordingly, the particular Nbs we analyzed did not contain a N-glycosylation consensus sequence (N-X-S/T) within their coding sequence. Referring to previous studies (Kubick et al., 2009) there is strong evidence that we observed signal peptide cleavage within the CHO cell-free system as we detected a single lower band for the cell-free synthesized signal peptide fused Nbs in SN2, but a double band in SN1. Signal peptide cleavage seemed to be almost complete for the translocated Nbs located in SN2, while in SN1 a mixture of cleaved and non-cleaved Nbs was detected (Figure 2). The observation that Nbs with cleaved signal peptide were detected in SN1 may be explained by the presence of small-sized microsomal vesicles which were not depleted during the preparation of fraction SN1 by centrifugation as previously reported by Thoring et al. (Thoring et al., 2017). In accordance to previous studies we observed that the translation efficiency of the cell-free system exceeded its capacity for protein translocation to the microsomal vesicles as a considerable amount of melittin signal peptide fused Nbs was detected in lysate fraction SN1 (Stech et al., 2017). For the future, it seems evident that for cell-free Nb synthesis CHO lysates can be used which are depleted from their endogenous microsomes. Alternatively, the lysate preparation procedure could be modified in a way, to quantitatively deplete the microsomes from the lysate.

The disulfide bond within the V_H_H framework region is evolutionally conserved and has been shown to be critical for the thermal stability of the V_H_H. However, it is not strictly required for maintaining antigen binding ability (Rudikoff and Pumphrey 1986; Pleiner et al., 2015). Several reports were published demonstrating that V_H_H retained antigen binding affinity even after removal of the canonical disulfide (Akazawa-Ogawa et al., 2016; Li et al., 2012; Liu et al., 2019). In addition, the production of functional, disulfide bond-independent Nbs (intrabodies) in the reducing environment of the *E. coli* cytoplasm for intrabody application has been demonstrated (Even-Desrumeaux et al., 2010; Olichon and Surrey 2007). The CDR3 of the anti-EGFR-Nb analyzed in this study is stabilized by van der Waals contacts and hydrogen bonds, but not by the interloop disulfide bond (Figure 1) which is frequently present in Nbs (Schmitz et al., 2013). The fact that the paratope of the anti-EGFR-Nb was found to be stable without the disulfide bond might be an explanation why this Nb was functional even without translocation to the microsomes. More difficult are conclusions on the meaning of disulfide bond formation within the anti-GFP-Nb. Kubala *et al.* revealed the structure of the GFP:GFP-Nb complex by X-ray crystallography, but due to the presence of the reducing agent DTT in the protein solution they found GFP-Nb with oxidized and reduced cysteines (Kubala et al., 2010). The CHO cell-free system used within this study was not specifically redox-optimized, but the reducing agent DTT was omitted from the coupled transcription-translation reaction. Clearly, a larger number of V_H_H candidates needs to be analyzed for a definite conclusion whether the conditions in the applied CHO cell-free system are sufficient to support disulfide bond formation within V_H_H in general. Balancing the redox potential in cell-free systems, e.g. by adding glutathione and/or disulfide bond isomerases and other chaperones to promote correct disulfide bond formation and isomerization, is common practice in eukaryotic (Bulleid et al., 1992; Kawasaki et al., 2003; Ezure et al., 2007; Stech et al., 2012; Martin et al., 2017) and prokaryotic (Ryabova et al., 1997; Bundy and Swartz 2011) cell-free systems. It is therefore very likely that these options will also be applicable to the CHO cell-free system used in this study. Based on the possibility to fine-tune the redox potential in open cell-free systems, it is conceivable that our system could be helpful to screen in particular for V_H_H which are intrinsically stable high affinity binders even in the absence of a disulfide bond which can be in particular useful for the *in vitro* evolution of intrabodies.

To summarize, here we demonstrate the proof-of-concept that soluble and functionally active Nbs can be synthesized using a CHO cell-free system. Due to the tremendously shortened time for Nb synthesis (in the batch-based system only 3 h) and the option for reaction parallelization, information on the binding activity of a multitude of different Nbs can be obtained within one day of work by using the proposed system. Thus, we present the CHO cell-free system as timesaving alternative to classical cell-based approaches. For the future we hypothesize that the system can become a valuable new source for *in vitro* display technologies enabling the *in vitro* selection of novel Nb sequences against desired targets. In particular, we envision the mammalian CHO cell-free system as a promising technology platform when eukaryotic and even mammalian systems are required to set up an effective screening and production pipeline for difficult-to-express Nb fusion proteins. The fruitful combination of *in vitro* display-based antibody selection technologies on cell-free synthesized difficult-to-express targets has been recently demonstrated (Nakai et al., 2022) emphasizing its great potential not only for antibody selection but also for difficult-to-express targets.

## Data Availability

The original contributions presented in the study are included in the article/Supplementary Material, further inquiries can be directed to the corresponding author.
